# Case Report: Complete tomographic resolution of focal choroidal excavation complicated with choroidal neovascularization after anti-VEGF treatment

**DOI:** 10.12688/f1000research.141099.1

**Published:** 2023-10-05

**Authors:** Imène Zhioua Braham, Selim Haddar, Mejdi Boukari, Manel Mokrani, Ilhem Mili, Raja Zhioua

**Affiliations:** 1 University of Tunis El Manar, faculty of medicine of Tunis, Tunis, Tunis, Tunisia; 2 Ophthalmology, Charles Nicolle University Hospital, Tunis, Tunis, Tunisia

**Keywords:** focal choroidal excavation, optical coherence tomography, choroidal neovascularization

## Abstract

Purpose

We report a case of focal choroidal excavation (FCE) that resolved after intravitreal injection of anti-vascular endothelial growth factor (VEGF) for choroidal neovascularization (CNV) and we describe its tomographic features.

Case report

A 43-year-old female presented with blurred vision and metamorphopsia in her left eye (LE) evolving for 10 years. The best corrected visual acuity (BCVA) was 20/20 in the right eye and 20/32 in the LE. Fundus examination revealed the presence of a yellowish foveal lesion which corresponded to a conforming FCE associated to a pachychoroid on swept-source optical coherence tomography (OCT). The OCT-Angiography showed a foveal flow void in the choriocapillaris layer corresponding to the FCE area.

Three years later, the patient complained of visual impairment, more metamorphopsia with a BCVA of 20/80 on her LE. The OCT showed intraretinal fluid with a foveal retinal pigment epithelium (RPE) detachment. The OCT-angiography confirmed the presence of CNV. Two months after one intravitreal bevacizumab injection, the OCT documented the complete resolution of macular edema, the regression of the CNV tissue and the restoration of a normal aspect of the fovea without any FCE. Her BCVA improved to 20/32 with resolution of the metamorphopsia. The OCT aspect remained stable during 3 years of follow-up.

Conclusion

CNV can develop in FCE and anti-VEGF therapy is a good option treatment. After treatment, FCE pattern can change et may completely resolve.

## Background

Focal choroidal excavation (FCE) is defined by a choroidal depression without evidence of posterior staphyloma or scleral ectasia, detected by swept-source optical coherence tomography (OCT) or by spectral-domain OCT using Enhanced Depth Imaging technology. ^1^ It is mainly an idiopathic clinical entity, suggested to be congenital, but can be also be secondary to other diseases. ^2^^–^^5^

Hereby, we report a case of foveal FCE complicated with choroidal neovascularization (CNV) which completely resolved after intravitreal injection of anti-VEGF.

## Objective

To describe the tomographic features of a case of FCE that resolved after intravitreal injection of anti-VEGF for CNV.

## Case report

**Figure 1.**
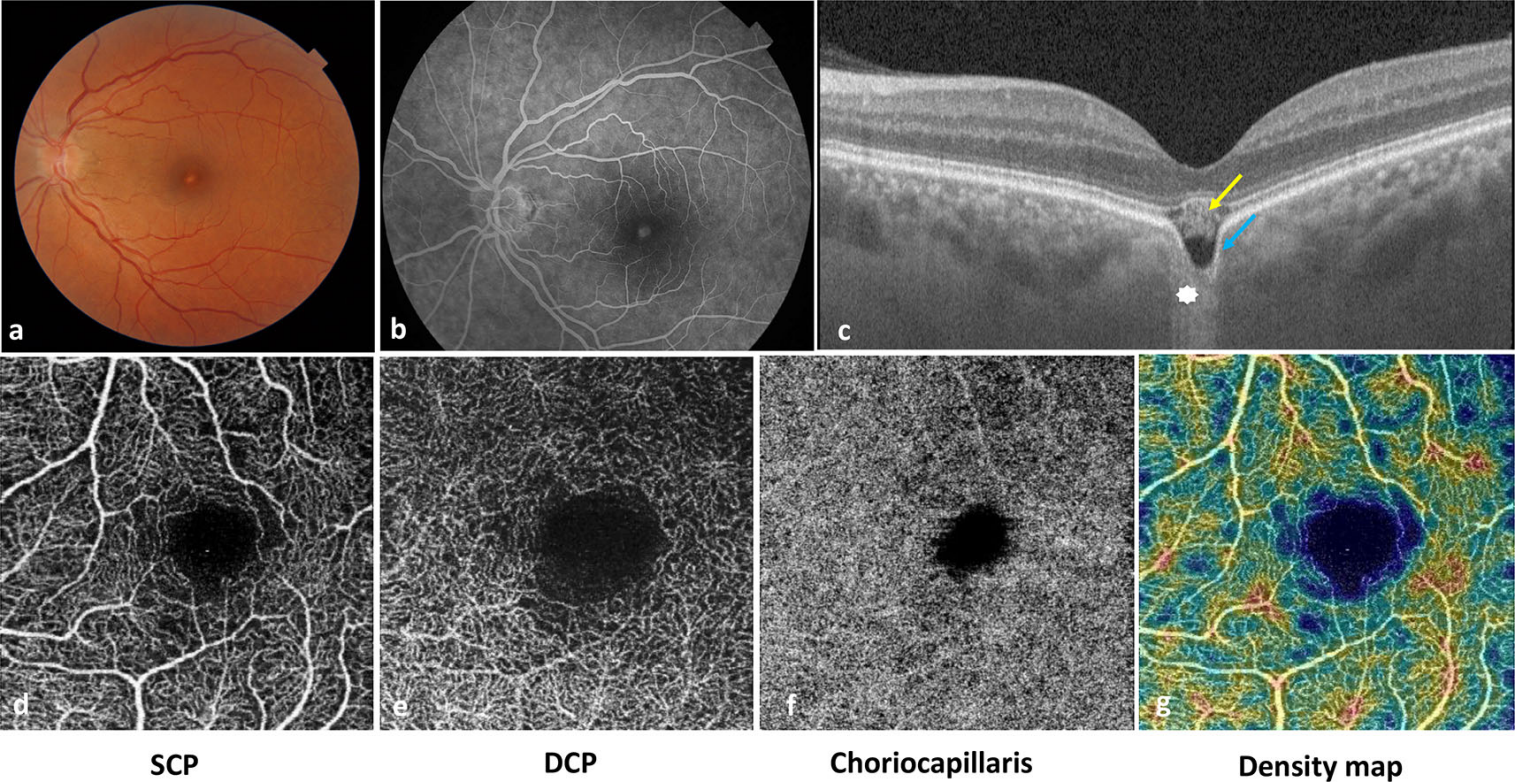
Multimodal imaging of a patient with focal choroidal excavation at initial presentation. a. Color fundus photography of the left eye showing a yellow plaque-like foveal lesion. b. Fluorescein Angiography showing foveal hyperfluorescence without leakage c. Swept-source OCT in the macular region showing a foveal depression of the choroid and the complex Bruch’s membrane-RPE (blue arrow). A localized foveal RPE atrophy was noted with a hypertransmission signal (white asterisk). The EZ was separated from the RPE by a hyperreflective material (yellow arrow) and a hyporeflective zone. The choroid appeared thinner in the area of the RPE depression than the parafoveal choroid which was a pachychoroid with pachyvessels. d.e.f.g. OCT-angiography showing a normal retinal vasculature with a foveal choriocapillaris flow void, corresponding to the thinned choroid beneath the FCE. OCT: optical coherence tomography, RPE: retinal pigment epithelium, EZ: ellipsoid zone, SCP: superficial capillary plexus, DCP: deep capillary plexus.

A 43-year-old female with no medical history presented with blurred vision and metamorphopsia in her left eye (LE) evolving for ten years. The best corrected visual acuity (BCVA) was 20/20 in the right eye and 20/32 in the LE (with a correction of -0,5 diopter). Anterior segment examination was unremarkable. Fundus examination revealed the presence of a yellowish foveal lesion ( Figure 1). The swept-source OCT showed a foveal depression of the choroid, the Bruch’s membrane and the retinal pigment epithelium (RPE). A localized foveal RPE atrophy was noted with a choroidal hyper-transmission signal on OCT. The EZ was separated from the RPE by a hyperreflective material and a hyporeflective zone. The choroid appeared thinner in the area of the depression than the parafoveal choroid which was a pachychoroid with pachyvessels. The OCT-Angiography showed a foveal flow void in the choriocapillaris layer corresponding to the FCE area. This multimodal imaging confirmed the diagnosis of a conforming FCE ( Figure 1). The right eye showed an isolated pachychoroid on OCT without FCE.

**Figure 2.**
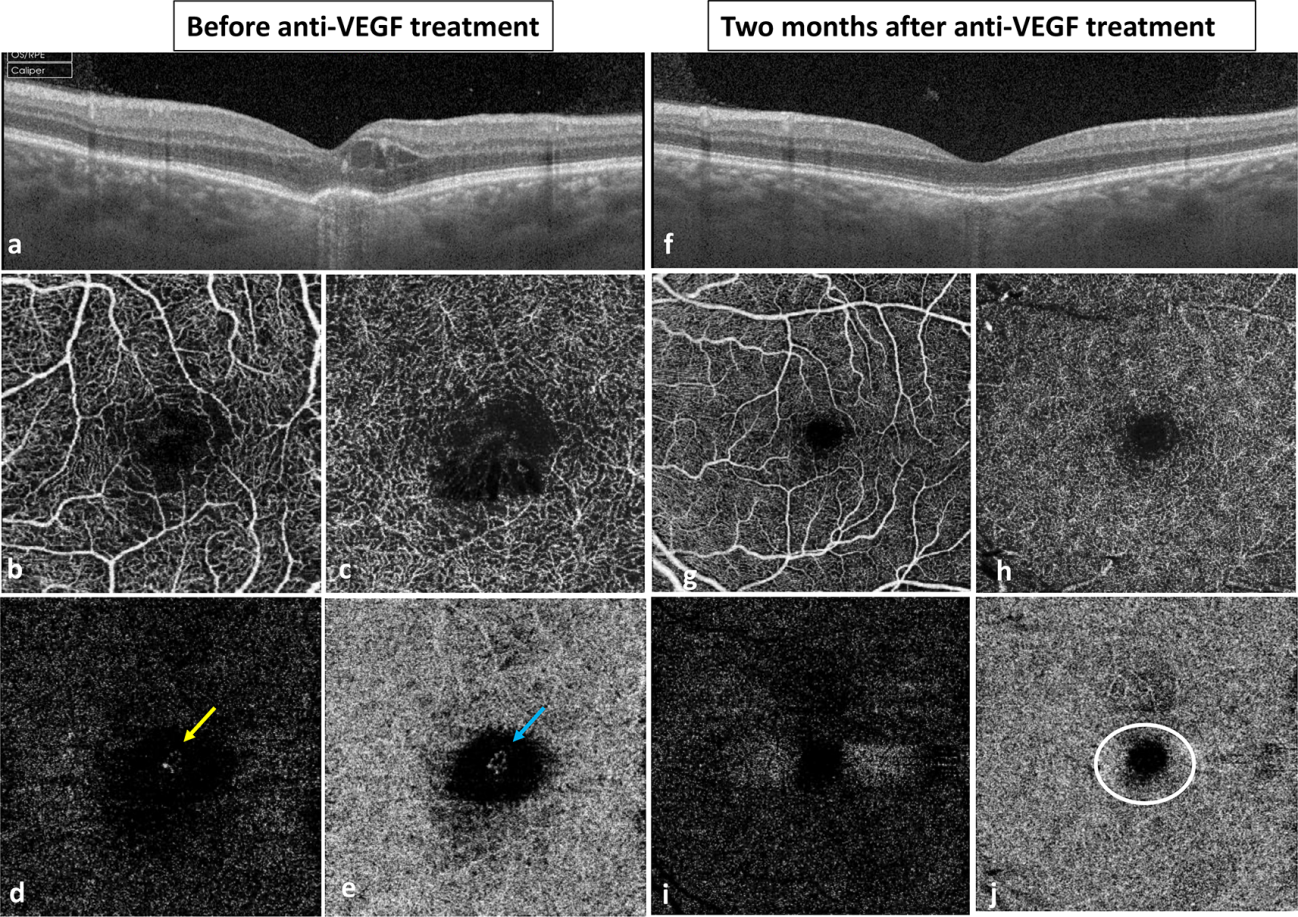
Imaging before and after anti-VEGF treatment in a patient with FCE complicated with CNV. a. Swept-source OCT showing intraretinal fluid with a foveal RPE detachment. b.c OCT-angiography at the level of the SCP (b) and the DCP (d) showing capillary disorganization and cystoid edema in the foveal region. d.e. OCT-angiography showing CNV at the level of the external retina (yellow arrow) and the choriocapillaris (blue arrow). f. Swept-source OCT two months after intravitreal injection of bevacizumab revealing normal aspect of the fovea with regular and intact RPE and resorption of the FCE. g.h. OCT-angiography at the level of the SCP (g) and the DCP (h) showing normalization of the retinal vasculature. i.j. Complete resorption of the CNV tissue on the external retina (i) and the choriocapillaris (j) on the OCT-angiography. The choriocapillaris showed persistence of the flow void in the foveal region corresponding to the localized choroidal thinning (white circle). VEGF: vascular endothelial growth factor, FCE: focal choroidal excavation, CNV: choroidal neovascularization, OCT: optical coherence tomography, RPE: retinal pigment epithelium, SCP: superficial capillary plexus, DCP: deep capillary plexus.

Three years later, the patient complained of visual impairment, more metamorphopsia with a BCVA of 20/80 on her LE. The OCT showed intraretinal fluid with a foveal retinal pigment epithelium (RPE) detachment within the excavation. The OCT-angiography confirmed the presence of CNV ( Figure 2). Two months after one intravitreal bevacizumab injection, the OCT documented the complete resolution of macular edema, the regression of the CNV tissue and the restoration of a normal aspect of the fovea without any FCE. Her BCVA improved to 20/32 with resolution of the metamorphopsia. The OCT aspect remained stable during three years of follow-up.

## Discussion

FCE is defined as an area of concavity in the choroid occurring in the absence of posterior staphyloma or scleral ectasia. Overlying retinal layers have usually a near-normal appearance. ^6^

The disease was first described par Jampol *et al.* in 2006 and was designated by the term FCE later in 2011 by Margolis et al. who also distinguished two tomographic entities: “conforming FCE” and “non-conforming FCE”. ^7^^,^^8^ OCT findings in “conforming FCE” show near-intact outer retinal layers laying against the excavated choroid without interruption and without any separation between photoreceptors and RPE. In “non-conforming FCE”, photoreceptors are separated from the underlying RPE within the excavated area with the presence of a hyporeflective space. Hyperreflective material may be visualized in this space indicating the presence of inflammatory material or degenerated outer segment residue. Additionally, Shinojima *et al.* described three patterns of the disease: the cone shaped, the bowl shaped and the mixed morphology. ^9^ Although foveal location is described in most of cases, a few cases of extrafoveal involvement have been reported, including the peripapillary location. ^10^

The exact pathogenesis of FCE remains to be understood. Usually, it is considered to be congenital, resulting from a focal defect of chorioretinal differentiation and found in asymptomatic eyes. Others may be acquired and secondary to an inflammatory or an ischemic process. ^11^ In fact, FCE can be associated with other ocular findings such as pachychoroid spectrum diseases (central serous chorioretinopathy, pachychoroid aneurysmal type 1 choroidal neovascularization), age related macular degeneration, chorioretinal inflammation, retinal dystrophies, choroidal and retinal tumors. ^12^^–^^14^ In our case, we noted a pachychoroid and pachyvessels on OCT in both eyes but without any sign of pachyhcoroid associated disease. In fact, choroidal vascular abnormalities have been described in association with FCE and included choroidal venous dilation (pachyvessels) and choroidal filling defects. ^15^ These findings suggest that local choroidal ischemia may affects the underlying RPE and outer retinal layers, leading in some cases to the development of a CNV. CNV is usually located within the excavation, as seen in our patient, or adjacent to the excavation. ^16^^,^^17^

Due to its minimal to absent visual impairment, uncomplicated FCE alone does not require treatment. Coexistent ocular findings such as CSC, PVC and CNV should be treated accordingly. The use of anti-VEGF is indicated in the treatment of CNV associated FCE with a good response. In our case, only one intravitreal injection was required, but authors reported one to four needed injections. ^17^ Following this treatment, conversion of non-conforming FCE with CNV to conforming pattern may be seen. ^18^ However, to our knowledge, this is the first report showing complete resorption of the excavation which disappeared after CVN treatment. The aspect of the RPE was restored and continuous, however the vasculature of the choroid remained affected on OCT-Angiography within the previous site of the depression.

In conclusion, FCE is well detected by OCT, which could be idiopathic or associated with other ocular diseases. Its relationship with pachychoroid has been reported but its physiopathology remains unknown. FCE can be complicated with CNV that develops within or near the margin of the excavation. Anti-VEGF treatment is a good option in case of CNV complicating FCE, but does not follow a standard protocol. The shape of FCE can change after CNV treatment and can also completely resolve.

## Consent

Written informed consent for publication of their clinical details and/or clinical images was obtained from the patient/parent/guardian/relative of the patient.

## Data Availability

All data underlying the results are available as part of the article and no additional source data are required. Figshare. CARE checklist and flowchart. DOI: https://doi.org/10.6084/m9.figshare.24117951.v1
