# Supplementary material for: Case Report: Complete tomographic resolution of focal choroidal excavation complicated with choroidal neovascularization after anti-VEGF treatment
Source: F1000Res. 2023 Oct 5;12:1270. [Version 1] doi: 10.12688/f1000research.141099.1 (PMC10955186; doi:10.12688/f1000research.141099.1)
Supplement: Peer review history

# Reviewer report: Reviewer response for version 1

**DOI:** 10.5256/f1000research.154513.r256520

**Published:** 2024-03-20

**Authors:** Niroj Kumar Sahoo

**Affiliations:** 1 Department of Retina and Vitreous, Anant Bajaj Retina Institute, L V Prasad Eye Institute, Vijayawada, India

The authors provide an interesting article on the response of FCE with CNVM to anti-VEGF. A few modifications are needed: 

 Case report section, Paragraph 1: The initial presentation looks like a "non-conforming" variant of FCE. This term can be included in the text.

 Figure 2: The presenting OCTA is a zoomed-in image/ 3 mm scan, while the follow-up image is a zoomed-out image/ 6 mm scan. It would be better to provide a zoomed-in OCTA image for the follow up too, for a better comparison of the retinal vasculature.

 Discussion: "However, to our knowledge, this is the first report showing complete resorption of the excavation which disappeared after CVN treatment. " We can still see the excavation on follow-up, although it has reduced in depth. This can be rephrased.

Are enough details provided of any physical examination and diagnostic tests, treatment given and outcomes?

Partly

Is the case presented with sufficient detail to be useful for other practitioners?

Yes

Is sufficient discussion included of the importance of the findings and their relevance to future understanding of disease processes, diagnosis or treatment?

Yes

Is the background of the case’s history and progression described in sufficient detail?

Yes

Reviewer Expertise:

imaging, macular disorders

I confirm that I have read this submission and believe that I have an appropriate level of expertise to confirm that it is of an acceptable scientific standard.

# Reviewer report: Reviewer response for version 1

**DOI:** 10.5256/f1000research.154513.r212716

**Published:** 2023-11-01

**Authors:** Imen Ksiaa

**Affiliations:** 1 University of Monastir, Monastir, Tunisia

This is a nice and well-documented case of focal choroidal excavation associated with the late development of choroidal neovascularization (CNV). A single intravitreal Bevacizumab injection resulted in the regression of the CNV lesion and resolution of the choroidal excavation. The data provided by the authors may be of clinical relevance.

 At the beginning of the discussion, authors should emphasize the originality of the case. Furthermore, the manuscript needs editing for typographical errors:

- In the abstract section
  - “Case report: A 43-year-old female presented...evolving for 10 years” might be changed to “A 43-year-old female presented with a 10-year history of blurred vision and…”;
  - “option treatment” to “treatment option”.
- In discussion section “…in 2006 and was designated by the term FCE” might be changed to “…was called FCE”.

Are enough details provided of any physical examination and diagnostic tests, treatment given and outcomes?

Yes

Is the case presented with sufficient detail to be useful for other practitioners?

Yes

Is sufficient discussion included of the importance of the findings and their relevance to future understanding of disease processes, diagnosis or treatment?

Yes

Is the background of the case’s history and progression described in sufficient detail?

Yes

Reviewer Expertise:

Medical retina, Surgical retina, Uveitis

I confirm that I have read this submission and believe that I have an appropriate level of expertise to confirm that it is of an acceptable scientific standard.
